# Variability of α/β ratios for prostate cancer with the fractionation schedule: caution against using the linear-quadratic model for hypofractionated radiotherapy

**DOI:** 10.1186/s13014-022-02010-9

**Published:** 2022-03-18

**Authors:** Ming Cui, Xian-Shu Gao, Xiaoying Li, Mingwei Ma, Xin Qi, Yuta Shibamoto

**Affiliations:** 1 Department of Radiation Oncology, Peking University First Hospital, Peking University, Beijing, People’s Republic of China; 2 Department of Radiation Oncology Gastrointestinal and Urinary and Musculoskeletal Cancer, Cancer Hospital of China Medical University, Shenyang, Liaoning People’s Republic of China; 3 Department of Radiology, Nagoya City University Graduate School of Medical Sciences, Nagoya, 467-8601 Japan

**Keywords:** LQ model, α/β ratio, Hypofractionated radiotherapy, High dose per fraction, Prostate cancer

## Abstract

**Background:**

Prostate cancer (PCa) is known to be suitable for hypofractionated radiotherapy due to the very low α/β ratio (about 1.5–3 Gy). However, several randomized controlled trials have not shown the superiority of hypofractionated radiotherapy over conventionally fractionated radiotherapy. Besides, in vivo and in vitro experimental results show that the linear-quadratic (LQ) model may not be appropriate for hypofractionated radiotherapy, and we guess it may be due to the influence of fractionation schedules on the α/β ratio. Therefore, this study attempted to estimate the α/β ratio in different fractionation schedules and evaluate the applicability of the LQ model in hypofractionated radiotherapy.

**Methods:**

The maximum likelihood principle in mathematical statistics was used to fit the parameters: α and β values in the tumor control probability (TCP) formula derived from the LQ model. In addition, the fitting results were substituted into the original TCP formula to calculate 5-year biochemical relapse-free survival for further verification.

**Results:**

Information necessary for fitting could be extracted from a total of 23,281 PCa patients. A total of 16,442 PCa patients were grouped according to fractionation schedules. We found that, for patients who received conventionally fractionated radiotherapy, moderately hypofractionated radiotherapy, and stereotactic body radiotherapy, the average α/β ratios were 1.78 Gy (95% CI 1.59–1.98), 3.46 Gy (95% CI 3.27–3.65), and 4.24 Gy (95% CI 4.10–4.39), respectively. Hence, the calculated α/β ratios for PCa tended to become higher when the dose per fraction increased. Among all PCa patients, 14,641 could be grouped according to the risks of PCa in patients receiving radiotherapy with different fractionation schedules. The results showed that as the risk increased, the k (natural logarithm of an effective target cell number) and α values decreased, indicating that the number of effective target cells decreased and the radioresistance increased.

**Conclusions:**

The LQ model appeared to be inappropriate for high doses per fraction owing to α/β ratios tending to become higher when the dose per fraction increased. Therefore, to convert the conventionally fractionated radiation doses to equivalent high doses per fraction using the standard LQ model, a higher α/β ratio should be used for calculation.

**Supplementary Information:**

The online version contains supplementary material available at 10.1186/s13014-022-02010-9.

## Background

With the development of high-precision radiotherapy, fractionation schedules to treat various tumors are changing [1]. Hypofractionated radiotherapy is being increasingly employed in clinics as stereotactic body radiotherapy (SBRT) and moderately hypofractionated intensity-modulated radiotherapy (IMRT), which have become valuable therapeutic approaches for a variety of tumors owing to the improved dose distribution. In addition, for prostate cancer (PCa), hypofractionated IMRT and SBRT seem to have radiobiological advantages based on the linear-quadratic (LQ) model estimation.

Since definitive hypofractionated radiotherapy is a relatively novel treatment, optimal dose fractionation schedules often need to be inferred from mathematical calculation, and an LQ model-based formula is frequently used to convert the conventionally fractionated radiation doses to high doses per fraction by clinicians due to its convenience and simplicity [2]. Recently, however, several investigators demonstrated that the standard LQ model may not be applicable to hypofractionated radiotherapy especially in SBRT [2–6], although other researchers insist that the LQ model can be used to estimate the antitumor effects of hypofractionated radiotherapy [7, 8].

The α/β ratio is a key factor in the LQ model [2]. Basically, the α/β ratio of a tumor is obtained from an in vitro dose-survival curve of tumor cells [2, 6], but this method cannot be applied to human tumors in patients. The α/β ratio can also be obtained from in vivo tumor or normal tissue responses to different fractionation schedules, and following this in vivo method, a mathematical method was elaborated to estimate the α/β ratio from clinical data employing various fractionation schedules [9, 10]. Using the method, the α/β ratios for various tumors have been reported, and PCa was found to have a low α/β ratio [9, 10], which was lower than the α/β ratio for normal tissue late reactions. Accordingly, moderately hypofractionated IMRT and SBRT are being increasingly used in the treatment of PCa. However, since the reliability of the LQ model in SBRT was questioned in recent studies [4–6], it may be necessary to re-evaluate the validity of converting conventionally fractionated doses to hypofractionated doses with the LQ model. Previous studies only tried to demonstrate that PCa has a low α/β ratio [9–12], and variability of the α/β ratios with the dose per fraction has not been investigated. Therefore, we carried out an analysis using an established mathematical calculation method to estimate the variation in the α/β ratio of PCa according to the daily fractional dose and to verify the applicability of the LQ model in hypofractionated radiotherapy.

## Methods

### Clinical data collection

We searched for relevant articles in PubMed with key words of “radiotherapy” or “radiation therapy” and “prostate cancer” or “prostatic carcinoma”. The inclusion criteria were as follows: (1) patients with PCa undergoing conventionally fractionated radiotherapy, moderately hypofractionated radiotherapy, or SBRT, with or without androgen deprivation therapy (ADT) and (2) 5-year biochemical relapse-free survival (5y-bRFS), number of patients, total dose, and fraction number or dose per fraction available from the articles. Articles that lacked the necessary fitting data or that used other fractionation schedules, such as hyperfractionated radiotherapy, were excluded. Conventionally fractionated radiotherapy was defined as that using 1.8–2.1 Gy per fraction. Moderately hypofractionated radiotherapy was defined as that using 2.19–3.5 Gy in our study. SBRT was defined as that using 6.5 Gy per fraction or greater according to National Comprehensive Cancer Network (NCCN) guidelines. Five-year bRFS according to the ASTRO or Phoenix definition was evaluated. The ASTRO definition of biochemical relapse is three consecutive rises in prostate-specific antigen (PSA) from the nadir [13]. The Phoenix definition of biochemical relapse is a rise of PSA over 2 ng/mL from the nadir [14]. Risk stratification of PCa was mostly made according to the NCCN guidelines risk group classification and in part D’Amico’s classification.

### Estimation of the α/β ratios

Statistical analyses were carried out exactly following the method of Miralbell and coworkers [15, 16]. Briefly, standard LQ models for tumor control at 5 years of the form:

1 $${\mathbf{P}} = {\mathbf{exp}}\left( { - {\mathbf{exp}}\left( {{\mathbf{k}} - {\mathbf{\alpha D}} - {\mathbf{\beta D}}^{2} /{\mathbf{N}}} \right)} \right)$$

or

2 $${\mathbf{P}} = {\mathbf{exp}}\left( { - {\mathbf{exp}}\left( {{\mathbf{k}} - {\mathbf{\alpha D}} - {\mathbf{\beta Dd}}} \right)} \right)$$

were fitted to the obtained data. In the formula, P is interpreted as the tumor control probability (5y-bRFS); D is the total dose; N is the number of fractions during the whole radiotherapy; d is the dose per fraction; and k represents the natural logarithm of an effective target cell number. α represents unrepairable lethal damage caused by a one-track action and β represents repairable sublethal damage caused by a two-track action in the DNA damage repair kinetics [17]. So, the α/β ratio can be considered as the balance between the two forms of damage.

Fitting was performed by maximum-likelihood methods and parameter estimates were obtained using the custom-written code in Stata version 12.0 [15, 16, 18]. The custom code is shown below:

- capture program drop myprog
- program myprog
- args lnf k d c rab
- quietly replace `lnf' = $ML_y1*$ML_y2*ln(exp(-exp(`k'-`d'-`c'/`rab'))) + $ML_y1*(1-$ML_y2)*ln(1-exp(-exp(`k'-`d'-`c'/`rab')))
- end
- constraint 1 D = C
- ml model lf myprog (k:All P =) (d:D, nocons) (c:C, nocons) (rab:), constraint(1)
- ml search
- ml maximize
- ml graph

The parameter estimation was directed by Professor Geng and Dr. Yin of the School of Mathematical Sciences, Peking University.

In order to reduce the errors of fitting parameters, we performed jackknife method. We removed one group every time from the 22 groups of data (taking conventionally fractionated radiotherapy group as an example); we could get 22 groups of sample data, which is C21 22in mathematics. Using these 22 groups of data, the average and standard error of the sample can be obtained, and then the population mean and the confidence interval of fitting parameters can be obtained. Moreover, we go a step further and calculate the Bias-Corrected and accelerated(BCa) intervals according to the Jung et al.’s methods[19]. For the parameters of the LQ model, significant figures were rounded to the 2nd decimal place.

In order to verify the accuracy of the results, we substituted the fitting results: k, α and α/β values and known parameters, i.e., total dose (D), single dose (d) and total fraction number (N), into the original TCP formula (1) or (2) and got a calculated P (5y-bRFS). Using goodness of fit test by chi-square test, the formula was $X^{2} = \sum\nolimits_{i = 1}^{k} {\frac{{\left( {O_{i} - T_{i} } \right)^{2} }}{{T_{i} }}}$, where *T*_*i*_ means theoretical frequency (original *P*) and *O*_*i*_ means observed frequency (calculated *P*). Then, we put calculated *X*^*2*^ into the critical value table and obtained corresponding *P* value. This method was checked whether there was statistical difference between the calculated P and the P from the original study. The statistical software SPSS 22.0 was used.

## Results

There were 45 articles (23,281 PCa patients) incorporated in this study published during 2003 to February 2021. Detailed information on the 45 articles is shown in Table 1. Among them, 5 articles reported on more than 1000 patients [20–24], and 3 reported on 500–1000 patients [25–27]. Among all 45 studies, a total of 38 articles (16,442 PCa patients) could be grouped according to fractionation schedules of radiotherapy and complete information could be extracted based on the inclusion and exclusion criteria; 15 were enrolled in the conventionally fractionated radiotherapy group, 24 were enrolled in the moderately hypofractionated radiotherapy group, and 8 were enrolled in the SBRT group. Nine articles were duplicated since the studies investigated both conventional fractionation and moderate hypofractionation. Detailed data from each article are shown in Tables 2, 3 and 4 according to the three different regimens of radiotherapy. To explore the relationship between α/β ratios and risks of PCa, we also divided each group into three subgroups by the risks in patients receiving radiotherapy with different fractionation schedules. Of all the 45 studies, 21 studies (14,641 PCa patients) could be grouped and the characteristics are shown in Additional file 1: Tables S1 to S3.

**Table 1:** Detailed information on 45 cited articles

Study	First author	Year	Journal^a^	Volume	Pages
1	Aizer	2009	Radiother Oncol	93	185–191
2	Alicikus	2011	Cancer	117	1429–1437
3	Cahlon	2008	IJROBP	71	330–337
4	Eade	2007	IJROBP	68	682–689
5	Kuban	2008	IJROBP	70	67–74
6	Lukka	2005	J Clin Oncol	23	6132–6138
7	Valdagni	2005	Radiother Oncol	75	74–82
8	Zelefsky	2008	IJROBP	71	1028–1033
9	Miralbell	2012	IJROBP	82	e17–24
10	Arcangeli	2012	IJROBP	84	1172–1178
11	Catton	2017	J Clin Oncol	35	1884–1890
12	Dearnaley	2016	The Lancet Oncology	17	1047–1060
13	Incrocci	2016	The Lancet Oncology	17	1061–1069
14	Kim	2014	Radiat Oncol J	32	187–197
15	Kupelian	2005	IJROBP	63	1463–1468
16	Leborgne	2009	IJROBP	74	1441–1446
17	Leborgne	2012	IJROBP	82	1200–1207
18	Pollack	2013	J Clin Oncol	31	3860–3868
19	Yeoh	2006	IJROBP	66	1072–1083
20	Cheung	2016	IJROBP	96	S33
21	Di Muzio	2016	Clin Oncol (R Coll Radiol)	28	490–500
22	Faria	2011	Radiother Oncol	101	486–489
23	Fonteyne	2012	IJROBP	84	e483–490
24	Hashimoto	2017	Int J Clin Oncol	NA	NA
25	Kuban	2010	IJROBP	78	S58–59
26	Kupelian	2007	IJROBP	68	1424–1430
27	Lieng	2017	Radiother Oncol	122	93–98
28	Livsey	2003	IJROBP	57	1254–1259
29	Mai	2010	IJROBP	78	S59
30	Patel	2013	IJROBP	86	534–539
31	Pervez	2017	Am J Clin Oncol	40	200–206
32	Shimizu	2017	Anticancer Res	37	5829–5835
33	Thomson	2012	Prostate Cancer	NA	NA
34	Viani	2016	Rep Pract Oncol Radiother	21	162–167
35	Bolzicco	2013	BMC Urol	13	NA
36	Fuller	2014	Front Oncol	4	NA
37	Kang	2011	Tumori	97	43–48
38	Katz	2013	Radiat Oncol	8	NA
39	King	2013	Radiother Oncol	109	217–221
40	Lee	2014	Medicine (Baltimore)	93	e290
41	Loblaw	2013	Radiother Oncol	107	153–158
42	Mantz	2014	Front Oncol	4	NA
43	Meier	2016	IJROBP	96	S33–34
44	Tsang	2021	Radiother Oncol	158	184–190
45	Chin,S	2020	IJROBP	107	288–296

*IJROBP* Int J Radiat Oncol Biol Phys, *NA* not available

^a^Journal abbreviations follow the PubMed style

**Table 2:** Conventionally fractionated radiotherapy group

Study number	Author	Number of patients	5y-bRFS	Total dose (D)	Fractions (N)/single dose	D^2^/N (C)	Definition of bRFS^b^
1	Aizer	352	0.748	75.6	42	136.08	P
2	Arcangeli	85	0.79	80	40	160	P
3	Catton	598	0.85	78	39	156	P
4	Dearnaley	1065	0.883	74	37	148	P
5	Eade	43	0.7	69	2.1	144.9	P
	Eade	552	0.81	72.5	2.1	152.25	P
	Eade	568	0.83	77.5	2.1	162.75	P
	Eade	367	0.89	81	2.1	170.1	P
6	Incrocci	397	0.771	78	39	156	P
7	Kim	56	0.641	70.2	39	126.36	P
8	Kuban	150	0.78	70	35	140	P
	Kuban	151	0.85	78	39	156	P
9	Kupelian	310	0.78	78	39	156	A
10	Leborgne	160	0.887	78	39	156	P
11	Lukka	470	0.4705	66	33	132	A
12	Pollack	152	0.852	76	38	152	P
13	Valdagni	161	0.7	74	37	148	A
14	Yeoh	109	0.555	64	32	128	A
15	Zelefsky	358	0.61	70.2	39	126.36	P
	Zelefsky	471	0.74	75.6	42	136.08	P
	Zelefsky	741	0.85	81	45	145.8	P
	Zelefsky	477	0.82	86.4	48	155.52	P

^b^*P* Phoenix, *A* ASTRO

**Table 3:** Moderately hypofractionated radiotherapy group

Study number	Author	Number of patients	5y-bRFS	Total dose (D)	Fractions (N)	D^2^/N (C)	Definition of bRFS^b^
1	Arcangeli	83	0.85	62	20	192.2	P
2	Catton	608	0.85	60	20	180	P
3	Cheung	230	0.837	67.5	25	182.25	P
4	Chin,S	112	0.68	52.5	20	137.8125	P
5	Dearnaley	1077	0.859	57	19	171	P
	Dearnaley	1074	0.906	60	20	180	P
6	Di Muzio	80	0.911	74.2	28	196.63	P
	Di Muzio	78	0.946	71.4	28	182.07	P
	Di Muzio	53	0.962	74.2	28	196.63	P
7	Faria	82	0.954	66	22	198	P
8	Fonteyne	113	0.94	56	16	196	P
9	Hashimoto	195	0.924	66	22	198	P
10	Lieng	96	0.81	60	20	180	P
	Lieng	27	0.88	66	22	198	P
11	Incrocci	407	0.805	64.6	19	219.64	P
12	Kim	30	0.929	70	28	175	P
13	Kuban	102	0.96	72	30	172.8	A
14	Kupelian	100	0.88	70	28	175	P
15	Kupelian	770	0.83	70	28	175	P
16	Leborgne	114	0.894	61.2	20	187.272	P
17	Mai	596	0.927	76.65	35	167.8635	P
18	Patel	129	0.97	66	22	198	P
19	Pervez	60	0.9167	67.5	25	182.25	P
20	Pollack	151	0.81	70.2	26	189.54	P
21	Shimizu	73	0.77	74.8	34	164.56	P
	Shimizu	21	0.92	74.8	34	164.56	P
	Shimizu	44	0.95	72.6	33	159.72	P
22	Thomson	30	0.5	57	19	171	P
	Thomson	30	0.58	60	20	180	P
23	Viani	149	0.946	69	23	207	P
24	Yeoh	108	0.574	55	20	151.25	A

^b^*P* Phoenix, *A* ASTRO

**Table 4:** SBRT group

Study number	Author	Number of patients	5y-bRFS	Total dose (D)	Fractions (N)	D^2^/N (C)	Definition of bRFS^b^
1	Bolzicco	100	0.944	35	5	245	P
2	Kang	44	0.936	34	4	289	P
3	King	1100	0.93	36.25	5	262.8	P
	King	385	0.925	35	5	245	P
	King	589	0.907	36.25	5	262.8	P
	King	126	0.958	39	5	304.2	P
4	Lee	45	0.897	36	5	259.2	P
5	Loblaw	84	0.98	35	5	245	P
6	Mantz	102	1	40	5	320	P
7	Meier	309	0.971	40	5	320	P
8	Tsang	43	0.92	36.25	5	262.8	P

^b^*P* Phoenix

Estimated α/β ratios are shown in Table 5. Among all 16,442 PCa patients, 7793 patients received conventionally fractionated radiotherapy, and the average α/β ratio was 1.78 Gy (95% confidence intervals (CI): 1.59–1.98, *P* < 0.001). There were 6822 patients in the moderately hypofractionated radiotherapy group. The α/β ratio was 3.46 Gy (95% CI 3.27–3.65, *P* < 0.001). In the SBRT group of 1827 patients, the α/β ratio was 4.24 Gy (95% CI 4.10–4.39, *P* < 0.001). We go a step further and calculate the BCa intervals of each groups:1.78 Gy (Bca 95% confidence intervals (CI): 1.62–1.97), 3.46 Gy (Bca95% CI 3.30–3.66), 4.24 Gy (BCa95% CI 4.14–4.34). Hence, the calculated α/β ratios for PCa tended to become higher when the dose per fraction increased. However, the k and α values were not affected by fractionation schedules. The k value was calculated as 5.35 (95% CI 4.61–6.08, *P* < 0.001), 1.15 (95% CI 0.21–2.09, *P* = 0.017), and 1.67 (95% CI − 4.80–8.15, *P* < 0.61), respectively, in patients receiving conventionally fractionated radiotherapy, moderately hypofractionated radiotherapy and SBRT. The α value was 0.043 Gy^−1^ (95% CI 0.029–0.056, *P* < 0.001), 0.026 Gy^−1^ (95% CI 0.016–0.036, *P* < 0.001), and 0.042 Gy^−1^ (95% CI − 0.27–0.36, *P* < 0.79), respectively.

**Table 5:** Parameters estimated with 95% CIs in different regimens of radiotherapy

	K			α (Gy^−1^)			α/β (Gy)		
	Estimate	95% CI	*P*	Estimate	95% CI	*P*	Estimate	95% CI BCa95% CI^*^	*P*
Conventional fractionation	5.35	4.61–6.08	< 0.001	0.043	0.029–0.056	< 0.001	1.78	1.59–1.98	< 0.001
								1.62–1.97	
Moderate hypofractionation	1.15	0.21–2.09	0.017	0.026	0.016–0.036	< 0.001	3.46	3.27–3.65	< 0.001
								3.30–3.66	
SBRT	1.67	− 4.80–8.15	0.61	0.042	− 0.27–0.36	0.79	4.24	4.10–4.39	< 0.001
								4.14–4.34	

^*^BCa 95% CI Bias-Corrected and accelerated intervals. We go a step further and calculate BCa intervals on the base of jackknife methods

Only 21 of 45 studies (14,641 PCa patients) could be grouped by the risks of PCa. For different risk subgroups, the results were shown in Table 6. At the same fractionation schedules, there were no practical clinical significance or significant differences in α/β values among the three risk groups. For example, the α/β ratios were 1.10 Gy (1.04–1.15, *P* < 0.001), 1.34 Gy (1.25–1.44, *P* < 0.001), and 0.39 Gy (0.33–0.45, *P* < 0.001) in the three risk groups, respectively, in the moderately hypofractionated radiotherapy group. We also calculated Bca intervals and the results were showen in Table 6. In the conventionally fractionated radiotherapy group, although the α/β ratios were significantly different among the three risk groups, there was no practical clinical significance. The calculated k value was 7.68 (95% CI 6.15–9.22, *P* < 0.001), 6.62 (95% CI 5.85–7.38, *P* < 0.001), and 4.93 (95% CI 4.00–5.87, *P* < 0.001), respectively, in the low-, intermediate-, and high-risk groups in the moderately hypofractionated radiotherapy group and the α value was 0.047 Gy^−1^ (95% CI 0.026–0.067, *P* < 0.001), 0.044 Gy^−1^ (95% CI 0.032–0.057, *P* < 0.001) and 0.011 Gy^−1^ (95% CI 0.0002–0.022, *P* = 0.046), respectively. According to the results, we found that the k and α values tended to decrease when the risks of PCa increased. In the conventionally fractionated radiotherapy group, the same conclusion could be drawn. In the SBRT groups, the α/β ratios were − 10.7 Gy (95% CI − 12.6– − 8.7, *P* < 0.001), 25.6 Gy (95% CI 21.6–29.6, *P* < 0.001), and 2.94 Gy (95% CI − 14.4–20.2, *P* = 0.74) in the low-, intermediate-, and high-risk groups, respectively. Since the α/β ratio in the low-risk patients was negative, we imposed non-negativity restrictions; thereafter, the α/β ratio in the low-risk group was 0.032 Gy (95% CI − 0.40–0.47, *P* = 0.89). The conclusion which came out from the conventionally fractionated radiotherapy group and moderately hypofractionated radiotherapy group could not be drawn in the SBRT group due to the limited number of articles involved.

**Table 6:** Parameters estimated with 95% CIs in different risks under different regimens of radiotherapy

Fractionation regimen	Risk group	K			α (Gy^−1^)			α/β (Gy)		
		Estimate	95% CI	*P*	Estimate	95% CI	*P*	Estimate	95% CI BCa 95% CI^*^	*P*
Conventional fractionation	Low risk	10.2	7.34–13.1	< 0.001	0.081	0.012–0.15	0.022	1.80	1.46–2.14	< 0.001
									1.56–2.11	
	Intermediate risk	8.20	6.85–9.56	< 0.001	0.073	0.041–0.10	< 0.001	2.29	2.12–2.47	< 0.001
									2.15–2.44	
	High risk	4.31	2.80–5.83	< 0.001	0.023	− 0.0053–0.051	0.11	0.98	0.91–1.05	< 0.001
									0.94–1.04	
Moderate hypofractionation	Low risk	7.68	6.15–9.22	< 0.001	0.047	0.026–0.067	< 0.001	1.10	1.04–1.15	< 0.001
									1.04–1.14	
	Intermediate risk	6.62	5.85–7.38	< 0.001	0.044	0.032–0.057	< 0.001	1.34	1.25–1.44	< 0.001
									1.26–1.43	
	High risk	4.93	4.00–5.87	< 0.001	0.011	0.0002–0.022	0.046	0.39	0.33–0.45	< 0.001
									.34–0.45	
SBRT^#^	Low risk	− 4.81	− 16.3–6.64	0.41	− 0.14	− 0.66–0.39	0.61	− 10.7	− 12.6– − 8.7	< 0.001
	Intermediate Risk	10.7	3.16–18.2	0.005	0.27	− 0.019–0.56	0.067	25.6	21.6–29.6	< 0.001
	High risk	16.0	− 42.7–74.8	0.59	0.14	− 0.89–1.17	0.79	2.94	− 14.4–20.2	0.74

^*^BCa 95% CI Bias-Corrected and accelerated intervals. We go a step further and calculate BCa intervals on the base of jackknife methods

^#^The BCa intervals could not be drawn in the SBRT group due to the limited number of articles involved

The preliminary results of verification of fitting results were shown in Table 7. The *X*^*2*^ were all < 1 in all three risk groups and the P values were all > 0.995 that meant there was no statistical difference between the calculated TCP and the TCP from the original study. In other words, our fitting was accurate.

**Table 7:** Preliminary results of verification of fitting results

Fractionation regimen	k	α (Gy^−1^)	α/β ratio (Gy)	X^2^	*P* value (goodness of fit test)
Conventional fractionation	5.35	0.043	1.78	0.10	> 0.995
Moderate hypofractionation	1.15	0.026	3.46	0.51	> 0.995
SBRT	1.67	0.042	4.24	0.01	> 0.995

In summary, for PCa patients receiving conventionally fractionated radiotherapy, moderately hypofractionated radiotherapy, and SBRT, the mean α/β ratios were 1.78, 3.46, and 4.24 Gy, respectively. Meanwhile, as the risks of PCa increased, the k and α values decreased.

## Discussion

The α/β ratio proposed in the early 1970’s derives from the LQ models [28, 29]. Factors that can influence α and/or β independently increase or decrease the α/β ratio. The major influencing factors are internal factors from cells themselves and external factors from physical or chemical effects [17, 30]. The internal factors include cell cycle regulation, cell repopulation, and DNA damage repair after irradiation. The external physical factors include temperature (hyperthermia), oxygenation (hypoxia), characteristics of radioactive rays-like linear energy transfer, and the dose rate. The external chemical factors are some anticancer drugs such as cisplatin, EGFR inhibitors, and PARP1 inhibitors. Thus, there are multiple factors that affect the α/β ratio and modify the radiosensitivity of tumors.

Our study showed that the α/β ratio tended to become higher when the dose per fraction increased. The α/β ratios may increase also dynamically during treatment, from approximately 4 Gy for ‘short’ fractionation schedules to about 1.5 Gy for long schedules, which probably reflects the process of accelerated repopulation in normal acute skin reactions [31, 32]. For late-responding tissues and slow-growing tumors like PCa, however, there may be no repopulation during radiotherapy [31], and the α/β ratio increase is not due to tumor cells repopulation. Also the time factors should not be considered in late-responding tissues [33, 34]. Thus, we did not take the time factor into consideration when converting doses using the standard LQ model.

Recent randomized trials demonstrated that hypofractionated radiotherapy was not superior to conventional radiotherapy in PCa. In the Radiation Therapy Oncology Group (RTOG) 0415 [35], Hypofractionated Irradiation for Prostate Cancer trial (HYPRO) [25], and the Fox Chase trial (ClinicalTrials.gov identifier: NCT00062309) [36], biological effective doses (BEDs) in hypofractionated vs. conventionally fractionated radiotherapy groups were calculated as 186.7 vs. 162.4 Gy, 211.0 vs 182.0 Gy, and 196.6 vs 177.3 Gy, respectively, using an α/β ratio of 1.5 Gy. All BEDs in the hypofractionated groups were 19–29 Gy higher than BEDs of the conventionally fractionated groups. Nevertheless, the higher BEDs did not lead to satisfactory improvements in the outcome. This may be attributable to the inaccurate conversion of radiation doses using the LQ model. In our study, the α/β ratio tended to become higher when the dose per fraction increased. When the doses in the three trials were converted with the LQ model using the α/β ratios that we estimated (1.78 Gy for conventionally fractionation and 3.46 Gy for moderate hypofractionation), BEDs of the hypofractionated and conventionally fractionated groups were 120.6 and 148.4 Gy, 128.1 and 165.6 Gy, and 125 and 161.4 Gy in the RTOG0415, HYPRO, and Fox Chase trial, respectively. The BEDs in the hypofractionated group were significantly lower than in the conventionally fractionated group. Thus, the non-superiority of the hypofractionated group could be in part explained by these BEDs calculated based on our results.

Several studies investigated the appropriateness of the LQ model at high doses per fraction. Previous in vitro and in vivo studies demonstrated that the LQ model overestimated the efficacy of tumor cell killing with a high dose per fraction [3, 37, 38]. Thus, several models were proposed modifying the standard LQ model to reasonably convert conventionally fractionated doses to equivalent single or hypofractionated doses. The lethal-potentially-lethal (LPL) model considered DNA lesion repair and could explain very effectively the shoulder on survival curves [39]; The modified LQ (MLQ) model made a better fit to the iso-effect data than the LQ model in a single high dose [40]. The “universal survival curve”(USC) model proposed by Park et al. combined two classical radiobiological models: the multitarget model and the standard LQ model that provide superior approximation of survival curves in the high-dose range. [41], and generalized LQ (gLQ) model encompasses the full dose range of possible dose delivery patterns and special radiotherapy schemes. [3]. Characteristics of these models have already been described [5]. Wang et al. [3] demonstrated that the problems in the LQ model derived from the amount of sublethal damage were reduced owing to conversion to lethal damage at a single high dose; if sublethal damage is converted to lethal damage, then the α/β ratio is elevated with a single high dose according to the definition of the α/β ratio. Other studies also revealed that cell death at high doses exceeded the probability of intracellular cell repair, and higher α/β ratios were shown with a linear survival curve [39, 42]. An in vivo study involving a murine tumor model demonstrated that an equivalent single high dose converted from fractionated radiotherapy was lower than the actual dose. However, when a higher α/β ratio was used, the discrepancy became smaller [38]. Our data agreed with their results. At different fractional doses, the α/β ratio tended to be higher when the dose per fraction increased (1.78 Gy for conventional fractionation, 3.46 Gy for moderate hypofractionation, and 4.24 Gy for SBRT). Especially in the SBRT groups, the high α/β ratio was marked.

We also found that α and k values decreased with risk elevation in the conventional fractionation and moderate hypofractionation groups. These results were similar to those in the previous study [15]. A decrease in the α values with escalation of the risk group can be attributed to higher radio-resistance of tumor cells in higher risk patients. k represents the natural logarithm of an effective target cell number, and a decrease in k values means that the effective target cell number is reduced with escalation of the risk group.

A recent publication was based on the dose distribution delivered to patients and provided another method for fitting parameter [43]. This method used the linear-quadratic Poisson TCP model with dose distribution like GTV (prostate gland), mpMRI‑GTV, D_50_ and dose volume histograms (DVH) to estimate α/β ratio and obtain solution space, initial parameter values, and optimal solution by optimizer. Our research approach was different from this method and used maximum likelihood principle in mathematical statistics to fit the α/β ratio according to the Miralbell model with tumor control probability (5y-bRFS), total dose, and number of fractions or dose per fraction; this method was much easier than the methods mentioned above.

The α/β ratio in the low-risk patients of the SBRT group was in the negative range. A study using external beam radiation therapy alone also had negative α/β ratios [44]. Repeated measures of PSA at 6 institutions were analyzed and data from 3 institutions including RTOG showed negative α/β ratios. In the Peter MacCallum Cancer Center, the α/β ratio was − 2.05 Gy (95% CI $- \infty - + \infty$). Another study found that the α/β ratio of arteriovenous malformation obliteration after radiosurgery was markedly negative (α/β =  − 49.3 ± 5.3) [45]. However, neither study explained why the α/β ratio was negative. These results as well as ours suggest a limitation of this calculation method in that it could possibly yield unrealistic α/β ratios, especially when the patient number is small. Although, we imposed non-negativity restrictions on the α/β ratio of the low-risk group (− 10.7 Gy to 0.032 Gy), it is merely a mathematical statistics method and may affect real result.

There are several limitations in our study. Since we divided the whole group into three subgroups according to the fractionation schedule, the dose ranges per fraction were relatively narrow in each fractionation group. This may increase the variability of the estimated α/β ratios, but we tried to solve this problem by including as many patients as possible. Subtle variations in patient evaluation including the definition of PSA failure and treatment including the dose prescription method among respective studies would also contribute to variability in the estimated α/β ratios; this problem is common to all studies of this kind, and is considered to be ameliorated by including a large number of patients. An analysis of over 14,000 patients showed that the derived α/β ratios were not different between studies using the ASTRO definition and those using the Phoenix definition [46]. The patient number in our study was larger than in any other studies investigating the α/β ratio for PCa. Also, the influence of ADT was not considered, as was the case with other previous studies, since a previous study indicated the minimal influence of ADT [15]. Another limitation is that our analysis was based on the prescription dose and the treatment outcome of the incorporated studies. Thus, 3D dose description or the DVHs were not incorporated into the analysis, because, for fitting parameters, we only needed prescription dose and treatment outcome according to TCP formula. Furthermore, the incorporated studies all used prescription dose which was widely used in clinical practice rather than dose distribution.

In conclusion, our study using mathematical statistics with 5y-bRFS data in PCa patients demonstrated that the α/β ratio was dependent on the fractionation schedule. In SBRT, the estimated α/β ratio was > 4 Gy. Therefore, to convert conventionally fractionated radiation doses to an equivalent single high dose, it may be necessary to use either a modified formula or a higher α/β ratio with the standard LQ model.

## Supplementary Information

**Additional file 1.** **Supplementary Table 1**. Conventionally fractionated radiotherapy in different risk groups.** Supplementary Table 2**. Moderately hypofractionated radiotherapy in different risk groups.** Supplementary Table 3**. SBRT in different risk groups.

## Data Availability

Not applicable.

## Abbreviations

PCa: Prostate cancer
LQ: Linear-quadratic
TCP: Tumor control probability
SBRT: Stereotactic body radiotherapy
IMRT: Intensity-modulated radiotherapy
ADT: Androgen deprivation therapy
5y-bRFS: 5-Year Biochemical Relapse-Free Survival
NCCN: National Comprehensive Cancer Network
BCa: Bias-corrected and accelerated
PSA: Prostate specific antigen
CI: Confidence intervals
RTOG: Radiation Therapy Oncology Group
BEDs: Biological effective doses
HYPRO: Hypofractionated Irradiation for Prostate Cancer Trial
LPL: Lethal-potentially-lethal
MLQ: Modified LQ
USC: Universal survival curve
gLQ: Generalized LQ
DVH: Dose volume histograms
