# Supplementary material for: Variability of α/β ratios for prostate cancer with the fractionation schedule: caution against using the linear-quadratic model for hypofractionated radiotherapy
Source: Radiat Oncol. 2022 Mar 18;17:54. doi: 10.1186/s13014-022-02010-9 (PMC8932192; doi:10.1186/s13014-022-02010-9)
Supplement: Supplementary file 1 — Additional file 1. Supplementary Table 1. Conventionally fractionated radiotherapy in different risk groups. Supplementary Table 2. Moderately hypofractionated radiotherapy in different risk groups. Supplementary Table 3. SBRT in different risk groups. [file 13014_2022_2010_MOESM1_ESM.docx]

**Supplementary Table 1 Conventionally fractionated radiotherapy in different risk groups**

| **Study number** | **Author** | **Number of low- risk patients** | **5y-bRFS of low- risk patients** | **Number of intermediate-**  **risk patients** | **5y-bRFS of intermediate- risk patients** | **Number of high-risk patients** | **5y-bRFS of high-risk patients** | **Total dose (D)** | **Fractions (N)** | **D^2^/N (C)** | **Definition of bRFS*^a^*** |
| --- | --- | --- | --- | --- | --- | --- | --- | --- | --- | --- | --- |
| 1 | Aizer | 80 | 0.853 | 155 | 0.822 | 117 | 0.622 | 75.6 | 42 | 136.08 | P |
| 2 | Alicikus | 49 | 0.98 | 89 | 0.89 | 32 | 0.7 | 81 | 45 | 145.8 | P |
| 3 | Cahlon | 100 | 0.98 | 192 | 0.85 | 186 | 0.7 | 86.8 | 48 | 156.24 | P |
| 4 | Dearnaley | 157 | 0.967 | 779 | 0.868 | 129 | 0.865 | 74 | 37 | 148 | P |
| 5 | Kim | 6 | 0.8 | 13 | 0.778 | 37 | 0.636 | 70.2 | 39 | 126.36 | P |
| 6 | Kuban | 31 | 0.89 | 71 | 0.88 | 48 | 0.55 | 70 | 35 | 140 | P |
|  | Kuban | 30 | 1 | 68 | 0.92 | 53 | 0.7 | 78 | 39 | 156 | P |
| 7 | Kupelian | 68 | 0.93 | 84 | 0.79 | 158 | 0.72 | 78 | 39 | 156 | A |
| 8 | Kupelian | 70 | 0.95 | 113 | 0.83 | 6 | 1 | 78 | 39 | 156 | P |
|  | Kupelian (ADT ＋) | 5 | 1 | 140 | 0.87 | 227 | 0.74 | 78 | 39 | 156 | P |
|  | Lukka | 113 | 0.66 | 278 | 0.38 | 79 | 0.28 | 66 | 33 | 132 | P |
|  | Miralbell | 57 | 0.87 | 118 | 0.67 | 50 | 0.32 | 74 | 37 | 148 | P |
|  | Yeoh | 34 | 0.76 | 63 | 0.57 | 12 | 0.42 | 64 | 32 | 128 | P |
| 9 | Leborgne | 56 | 0.98 | 66 | 0.84 | 8 | 0.87 | 78 | 39 | 156 | P |
| 10 | Valdagni | 75 | 0.845 | 58 | 0.613 | 28 | 0.653 | 74 | 37 | 148 | A |

*a: Abbreviations:* P = Phoenix; A = ASTRO.

**Supplementary Table 2 Moderately hypofractionated radiotherapy in different risk groups**

| **Study number** | **Author** | **Number of low-risk patients** | **5y-bRFS of low-risk patients** | **Number of intermediate- risk patients** | **5y-bRFS of intermediate- risk patients** | **Number of high-risk patients** | **5y-bRFS of high-risk patients** | **Total dose (D)** | **Fractions**  **(N)** | **D^2^/N**  **(C)** | **Definition of bRFS*^a^*** |
| --- | --- | --- | --- | --- | --- | --- | --- | --- | --- | --- | --- |
| 1 | Dearnaley | 164 | 0.966 | 784 | 0.902 | 126 | 0.842 | 60 | 20 | 180 | P |
|  | Dearnaley | 163 | 0.909 | 784 | 0.86 | 130 | 0.783 | 57 | 19 | 171 | P |
| 2 | Fonteyne | 45 | 0.98 | 50 | 0.94 | 18 | 0.83 | 56 | 16 | 196 | P |
| 3 | Hashimoto | 27 | 1 | 70 | 0.932 | 98 | 0.898 | 66 | 22 | 198 | P |
| 4 | Kim | 5 | 1 | 5 | 1 | 20 | 0.885 | 70 | 28 | 175 | P |
| 5 | Kupelian | 262 | 0.94 | 216 | 0.83 | 292 | 0.72 | 70 | 28 | 175 | P |
| 6 | Kupelian | 36 | 0.97 | 30 | 0.93 | 34 | 0.75 | 70 | 28 | 175 | P |
| 7 | Leborgne | 29 | 0.96 | 45 | 0.84 | 15 | 0.85 | 61.2 | 20 | 187.27 | P |
| 8 | Mai | 226 | 0.969 | 264 | 0.933 | 106 | 0.82 | 76.65 | 35 | 167.86 | P |
| 9 | Livsey | 181 | 0.82 | 247 | 0.56 | 277 | 0.39 | 50 | 16 | 156.25 | A |
| 10 | Viani | 51 | 1 | 55 | 0.964 | 43 | 0.86 | 69 | 23 | 207 | P |
| 11 | Logue (ADT +) | 111 | 0.69 | 323 | 0.57 | 412 | 0.53 | 50 | 16 | 156.25 | P |
|  | Logue | 311 | 0.79 | 516 | 0.63 | 409 | 0.59 | 50 | 16 | 156.25 | P |
|  | Lukka | 113 | 0.59 | 265 | 0.47 | 88 | 0.29 | 52.4 | 20 | 137.29 | P |
|  | Miralbell | 21 | 0.9 | 30 | 0.72 | 20 | 0.74 | 56 | 14 | 224 | P |
|  | Yeoh | 26 | 0.73 | 57 | 0.67 | 25 | 0.64 | 55 | 20 | 151.25 | P |
|  | Kupelian (ADT +) | 59 | 0.95 | 210 | 0.84 | 213 | 0.65 | 70 | 28 | 175 | P |
|  | Kupelian | 198 | 0.95 | 108 | 0.84 | 4 | 0.65 | 70 | 28 | 175 | P |

*a: Abbreviations:* P = Phoenix; A = ASTRO.

**Supplementary Table 3 SBRT in different risk groups.**

| **Study number** | **Author** | **Number of low-risk patients** | **5y-bRFS of low-risk patients** | **Number of intermediate- risk patients** | **5y-bRFS of intermediate- risk patients** | **Number of high-risk patients** | **5y-bRFS of high-risk patients** | **Total dose (D)** | **Fractions (N)** | **D^2^/N (C)** | **Definition of bRFS*^a^*** |
| --- | --- | --- | --- | --- | --- | --- | --- | --- | --- | --- | --- |
| 1 | Fuller | 40 | 0.98 | 39 | 0.92 | NA | NA | 38 | 4 | 361 | P |
| 2 | Kang | 5 | 1 | 10 | 1 | 29 | 0.908 | 34 | 4 | 289 | P |
| 3 | Katz | 211 | 0.97 | 81 | 0.907 | 12 | 0.741 | 36 | 5 | 259.2 | P |
| 4 | King | 641 | 0.952 | 334 | 0.841 | 125 | 0.812 | 36.25 | 5 | 262.81 | P |
|  | King | 254 | 0.958 | 108 | 0.723 | 23 | NA | 35 | 5 | 245 | P |
|  | King | 319 | 0.95 | 188 | 0.872 | 82 | 0.741 | 36.25 | 5 | 262.81 | P |
|  | King | 68 | 0.944 | 38 | 0.967 | 20 | NA | 39 | 5 | 304.2 | P |
| 5 | Meier | 172 | 0.973 | 137 | 0.971 | NA | NA | 40 | 5 | 320 | P |

*a: Abbreviations:* P = Phoenix; A = ASTRO
